# Antifouling Copper Surfaces Interfere with Wet Chemical Nitrate Sensors: Characterization and Mechanistic Investigation

**DOI:** 10.1021/acsestwater.4c00749

**Published:** 2024-12-17

**Authors:** Adrian M. Nightingale, Alexander D. Beaton, Antony J. Birchill, Sharon Coleman, Gareth W. H. Evans, Sammer-ul Hassan, Matthew C. Mowlem, Xize Niu

**Affiliations:** † Mechanical Engineering, Faculty of Engineering and Physical Sciences, University of Southampton, Southampton, SO17 1BJ, United Kingdom; ‡ Ocean Technology and Engineering Group, National Oceanography Centre, Southampton, SO14 3ZH, United Kingdom

**Keywords:** Griess, copper, thiol, interference, vanadium, cadmium column

## Abstract

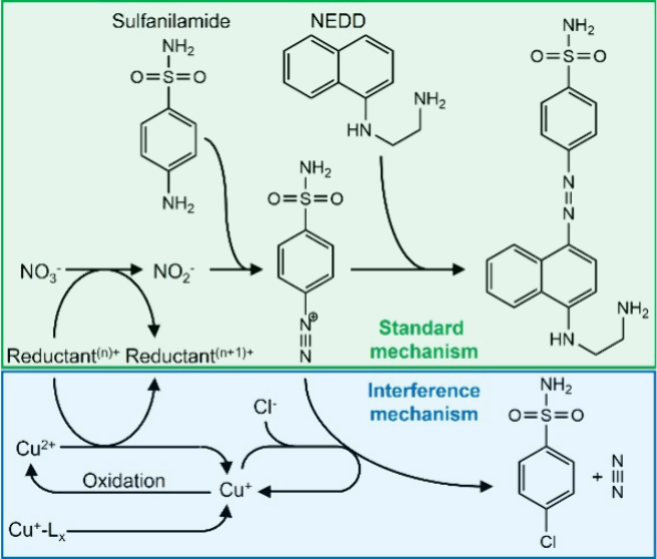

Wet chemical sensors autonomously sample and analyze water using chemical assays. Their internal fluidics are not susceptible to biofouling (the undesirable accumulation of microorganisms, algae, and animals in natural waters) due to the harsh chemical environment and dark conditions; however, the sample intake and filter are potentially susceptible. This paper describes the use of copper intake filters, incorporated to prevent fouling, on two different wet chemical nitrate sensors that each use different variants of the Griess assay (in particular, different nitrate reduction steps) to quantify nitrate concentrations. When the copper filters were used, measurements were perturbed in both sensors. Here we describe how the interference was first encountered in field testing and how it was subsequently replicated in laboratory testing. We show how the interference is due to the presence of copper ions from the filters and propose a mechanism for how it interferes with the assay, accounting for differences between the different versions of the Griess assay used in each sensor, and discuss strategies for its management. The findings are not just limited to wet chemical sensors but also more broadly applicable to any laboratory nitrate or nitrite analysis based on the Griess assay.

## Abstract 2

Copper surfaces can prevent fouling in aquatic environments, but their presence adversely effects wet chemical nitrate sensors due to dissolved copper ions interfering with the Griess assay.

## Introduction

*In situ* sensors allow continuous monitoring of the aquatic environment with much increased temporal and spatial resolution compared to traditional spot-sampling and lab analysis. Wet chemical sensors implement established laboratory assays into integrated field-deployable systems that can automatically sample and analyze the water. Biofouling—the accumulation of microorganisms, algae, and animals on submerged surfaces—is an inherent problem for all structures submerged in natural waters, most notably in warm nutrient-rich waters. Left unchecked, biofouling can perturb sensor data and hence is an important consideration when planning sensor deployments.^1^ Macrofoulers can obstruct water flow to sensors, while biofilms can coat surfaces, such as conductivity cells and optodes, reducing measurement quality.^2−4^ Many antifouling approaches have been developed with no one strategy providing a universal solution.^4^ Passive strategies such as volumetric biocide, which release antifouling compounds (e.g., organotin or copper) into the microlayer above the coated surface to prevent or slow biofouling,^5^ are advantageous as they do not require external energy. For example, several Sea-Bird CAT instruments can be deployed with a plastic antifouling device containing bis(tributyltin) oxide (AF24173 Anti-Foulant Device), while the SUNA UV nitrate sensor can be equipped with a copper antifouling guard.^6^ Similarly, surface biocide approaches involve constructing the sensing area from materials with antifouling properties, for instance, coating optodes with transparent polymers doped with surfactants.^7,8^ Active strategies offer more thorough cleaning but require external energy. Examples include retracting sensitive elements into an inert or biocide chamber, flow-through systems with copper tubing, using copper shutters to cover sensing surfaces between measurements, ultraviolet radiation, electrochemical generation of toxic substances, and mechanical cleaning of surfaces using wipers.^9−12^

**Figure 1:**
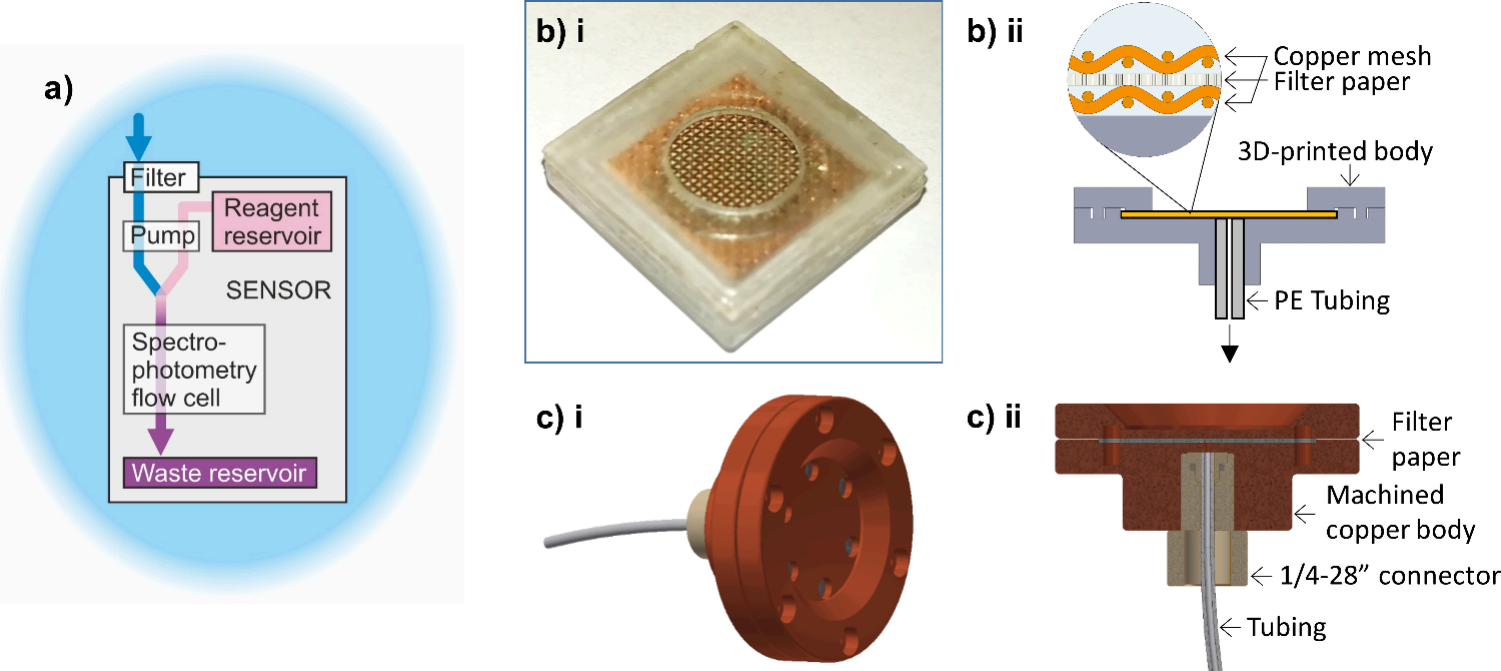
a) Cartoon illustrating the general mode of operation of wet chemical nitrate sensors, whereby a water sample is pulled into the sensor through a filter and mixed with a colorimetric reagent, and the resulting color quantified via spectrophotometry. b) The filter trialled by the UoS group, featuring Cu mesh sandwiching a filter membrane, shown in a photograph (i) and as a schematic cross section interfaced with polyethylene (PE) tubing (ii). c) The filter trialled by NOC, shown in two projections.

Our two research teams have separately developed microfluidic sensors to measure nitrate and nitrite concentrations *in situ* using the colorimetric Griess assay.^13−19^ While they use slightly different flow regimes (single-phase stop flow versus continuous droplet flow) they both operate in a broadly similar fashion (Figure 1)—drawing water from the external environment into the system through a filter and then flowing it onward into micro milled channels or narrow bore tubing where it mixes with reagents (different variations of the Griess assay) to generate a quantifiable color. Biofouling of internal channels would increase fluidic resistance and alter the composition of the sampled water; however, the darkness combined with the harsh chemicals within the Griess assay creates an inhospitable environment that prevents biofilm formation.^20^ The sensors’ external surfaces, including the sample intake and filter, are still potentially susceptible to biofouling under suitable conditions however.

To prevent any potential fouling on the sensors’ intake filter, groups at the University of Southampton (UoS) and the UK’s National Oceanography Centre (NOC) separately developed intake filters featuring antifouling copper (Cu) surfaces. These were independently tested in field trials on different nitrate sensors, but both trials delivered unexpectedly lower measurements. Here we describe these trials and subsequent collaborative work to determine the underlying mechanism of the measurement perturbation. While the underlying mechanism for each sensor is the same, there are some differences, corresponding to the different reductants that are featured in the colorimetric assay used in each sensor.

## Experimental Section

### Sensors

The two sensors used in this study have been described in detail elsewhere.^13−15,21^ Both operate on the same basic underlying principle, whereby water samples are brought into the sensor and measured using two different variations of the Griess colorimetric assay (Figure 1a) and are briefly described below.

The NOC total nitrate microfluidic sensors are composed of a three layer poly(methyl methacrylate) chip with precision milled micro channels (150 μm wide, 300 μm deep), mixers, and optical components consisting of light emitting diodes and photodiodes. Electronics, valves, and syringe pumps are mounted on the chip, which is encased in a dark watertight PVC tube. The sensor has an off-chip copperized cadmium column^22^ which reduces NO_3_^–^ to NO_2_^–^. The nitrite subsequently reacts with the reagent to generate a colored product. The analytical procedure measures combined nitrite and nitrate (which in oxygenated waters will be primarily nitrate). The sensor procedure used here was as follows: 69 μL of sample, blank, or standard solution was injected into the chip along with 69 μL of imidazole buffer. The solutions mixed in a serpentine mixing channel and then entered the off-chip copperized cadmium column. This mixture was flushed through the chip to a waste reservoir, and this procedure was repeated four times to fully flush the chip and prevent carryover from previous measurements. On the fifth flush, 69 μL of Griess reagent was added downstream of the copper coated cadmium column and the resulting mixture left in spectrophotometric measurement cells for 55 s to allow mixing, reaction, and hence color development. Each sample measurement was accompanied by measurement of a blank solution and a standard solution to give a fully calibrated measurement, which took a total of 19 min. The limit of detection of the sensor, defined as three times the standard deviation of a 0.05 μM nitrate standard, has been reported as 25 nM,^13^ several orders of magnitude below the concentration of samples analyzed in this study.

The UoS nitrate and nitrite sensor operates under a droplet flow regime whereby water samples are analyzed as discrete droplets carried by a stream of immiscible oil (with the oil also acting as a quasi-blank for the optical measurement). Water is drawn into the sensor by a peristaltic pump, passing through the pump and on to a T-junction where a reagent, composed of a vanadium reducing agent (VCl_3_) and Griess reagent, is introduced. The mixture is then broken into a stream of droplets as it meets a flow of fluorinated oil (Fluorinert FC-40). The droplet generation dynamics (frequency, droplet size) are controlled via our previously reported peristaltic method whereby a single droplet is robustly generated with each cycle of the peristaltic pump.^23^ The droplet contents mix as they are carried downstream and react to produce a colored product, which is quantified by two absorption flow cells. The flow cells are positioned before and after an internal heater, allowing the sensor to quantify both nitrite (before the heater has driven the nitrate reduction step) and combined nitrite and nitrate (after the heater). The sensor used for this particular work was an earlier prototype^21^ of the sensor we reported in 2019,^15^ with the major difference being the use of onboard standard: The prototype sensor here interspersed standard droplets within the sample droplets,^21,24^ whereas in the mature sensor the sample inflow was periodically swapped using a valve to the standard. The prototype featured no major changes compared to the mature sensor, which has a limit of detection of 1.7 μM (2 orders of magnitude lower than the measurements in this study), and consumes reagent at 2.8 mL/day when continuously measuring at 0.1 Hz.^15^

### Filter Description

The UoS’s custom-made filter (Figure 1b) was composed of a 3D printed body (“VeroClear” material, Objet500 Connex3 polyjet printer) which held together standard filter material sandwiched on either side by copper mesh (size 50 mesh: wire diameter ∼135 μm, gap ∼295 μm). The copper mesh on the outer side was included to prevent biological growth on the filter surface, while the mesh on the inner side also ensured that the filter could not lie flat against the filter body, so there would always be a clear fluidic path through to tubing attached to the filter body.

The NOC’s custom-made filter (Figure 1c) was composed of two parts, machined from solid copper, that sandwiched standard filter material. For comparison, this was tested versus a 0.45 μm poly(ether sulfone) syringe filter (Millex HP, MERCK, Millipore, U.S.A.).

### Sensor Deployments

#### NOC Field Deployment

Two sensors were deployed for 5 days in January 2018 on the same mooring in Southampton Water estuary (Empress Docks) at approximately 0.5 m depth; one sensor was fitted with a plastic Luer lock filter and one with a custom-made copper filter. Measurement times for both sensors were synchronized to ensure simultaneous hourly measurements.

#### UoS Field Deployment

The same UoS sensor was deployed twice for 24 h in the summer of 2017. In each case, it was suspended from a pontoon (∼30 cm depth) on the tidal River Itchen, approximately 250 m downstream of the Woodmill tidal barrier and approximately 7 km upstream from the NOC deployment location. For the first deployment no filter was used on the water inlet, and for the second the prototype copper filter was used.

### UoS Assay Testing of Nitrate Exposed to Copper Mesh

Three mL of a 300 μM nitrate or nitrite standard was added into each well of a 6-well plate. Five approximately 2 cm × 2 cm squares of copper mesh (the same material used in the UoS filter) were cut out and weighed to ensure they had the same approximate mass (within 2% of the average). Each mesh square was placed into a well with the sixth well left as a control. Each mesh was removed at a prespecified time between 30 s and 10 min.

Having been exposed to the copper mesh, the water samples were analyzed by taking a 1 mL sample and adding in 1 mL of the UoS Griess reagent (with or without VCl_3_) in a 24-well plate. The well plate was placed in a 65 °C oven for 15 min and then removed and left to cool for a further 15 min. The resulting colorimetric response was analyzed using a plate reader (BMG Labtech FLUOstar Omega).

### UoS Assay Testing of Samples Exposed to in the Lab with Cu(II) Spiking

In a 24-well plate, 0.45 mL of a 300 μM nitrate or nitrite standard solution was added into six wells, followed by 0.1 mL of a Cu(II) standard solution (each well receiving a separate standard, from 0 to 10 mM). After leaving for 5 min 0.42 mL of the Griess reagent with VCl_3_ was added. The well plate was placed in a 65 °C oven for 15 min and then left to cool for a further 15 min. Finally analysis was completed using a plate reader (BMG Labtech FLUOstar Omega). All measurements were repeated in triplicate, so that 18 of the 24 wells were used.

### NOC Sensor Lab Testing with Cu(II) Spiking and Artificial Estuarine Water

Testing with the NOC sensor used 100 μM nitrate and nitrite solutions spiked with copper concentrations identical to those used for the UoS assay testing. The lab-on-chip sensor system was identical to that recently described.^25^ The copper-spiked nitrate and nitrite solutions were analyzed by the sensor with the cadmium reduction tube fitted, and the nitrite solutions were also tested in the absence of the cadmium reduction tube, where it was replaced by a length of 0.5 mm internal diameter PTFE tubing.

### Inductively Coupled Plasma-Optical Emission Spectrometer Analysis

Water and artificial seawater from the filter time course experiments were acidified with trace metal grade nitric acid (HNO_3_ Fisher Scientific, Primar Plus) to a final concentration of 2%. The leachate was analyzed by inductively coupled plasma-optical emission spectrometry (ICP-OES; SPECTRO ARCOS). The instrument was calibrated using a series of external standards made by serial dilution of a copper containing multielement standard in 2% nitric acid (HNO_3_).

## Results

**Figure 2:**
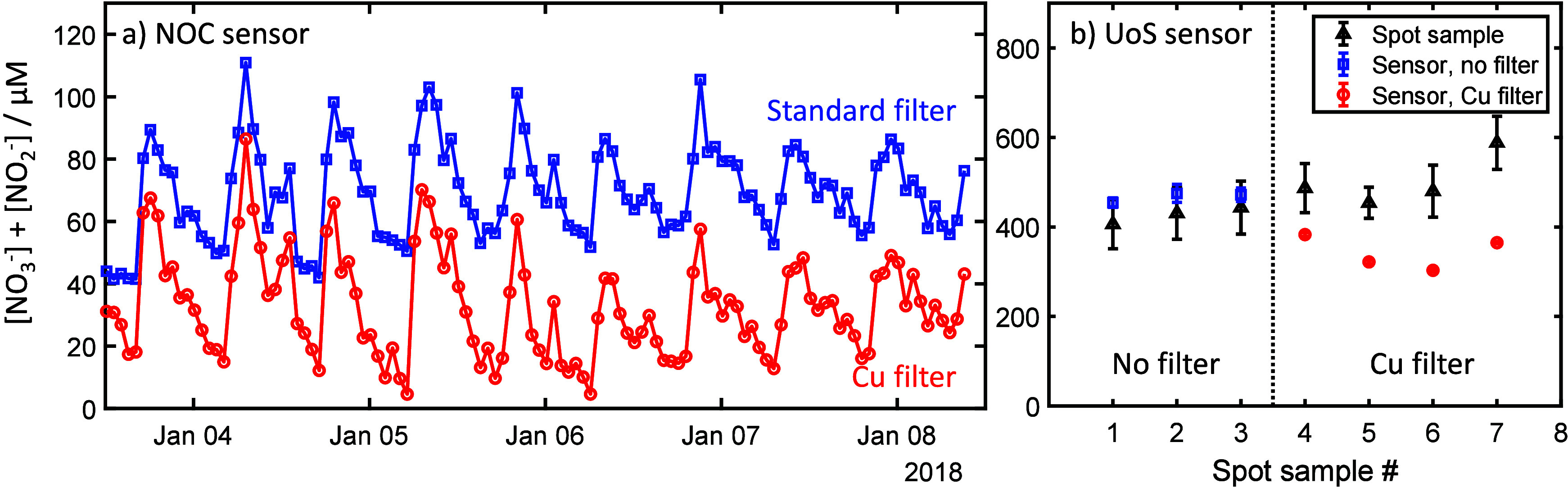
Field data from sensors exhibiting interference from Cu filters. A) Data from two NOC sensors simultaneously deployed side-by-side in the estuarine River Itchen, one with a Cu filter and one with a standard syringe filter. B) UoS data showing spot-samples compared with the corresponding sensor data for a sensor deployed in the River Itchen without and with a copper filter (samples 1–3 vs 4–7, respectively).

The copper interference first became apparent during two independent sensor deployments carried out by the NOC and the University of Southampton during 2017 and 2018. In the NOC’s test, two identical sensors were deployed side by side in Empress docks, Southampton where the River Itchen meets Southampton Water, a tidal estuary fed by the Rivers Itchen and Test. They were left unattended sampling hourly for 5 days. One sensor featured a standard syringe filter, previously used in multiple successful deployments,^13,17−19^ while the other used the experimental copper filter. The standard filter sensor gave nitrate results in keeping with previous measurements for this location^13^ (Figure 2a), with a characteristic periodic variation consistent with the tidal shift between high nutrient fresh water and low nutrient marine water. The copper filter sensor showed similar overall trends but with absolute values 20–90% lower than the sensor with the standard filter. The relative undermeasurement of samples was more pronounced when the nitrate concentrations were at the troughs of the periodic variation when seawater would be predominating.

In the UoS group’s test, a prototype sensor was deployed in the tidal River Itchen for 24 h on two occasions, 2 weeks apart, with spot samples taken for comparison. In the first deployment, no filter was used, while the copper filter was used in the second. As was the case for the NOC sensor a characteristic tidal signal was seen in the nitrate measurements (data not shown), but a discrepancy was seen when comparing sensor data to manual spot samples taken at the same time (Figure 2b). When no filter was used, the corresponding sensor data (blue squares) matched the spot samples (black triangles) well, but when the copper filter was used (red circles), there was a negative bias of 20–40%. Later deployments at the same location of a more mature prototype which utilized nylon mesh filters, showed excellent agreement between the sensor and spot samples.^15^

**Scheme 1:**
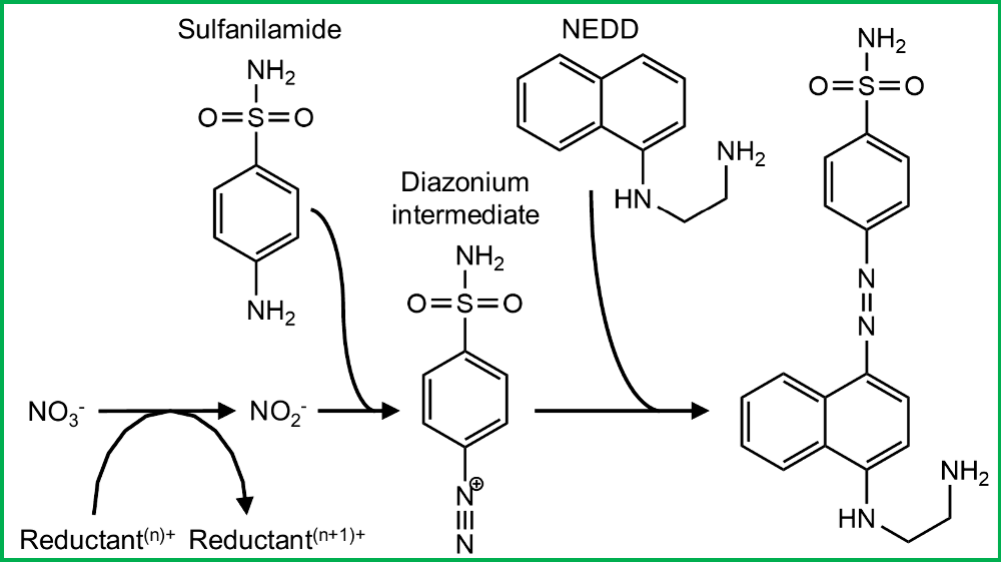
Standard Reaction Mechanism for the Colorimetric Nitrate Assay

Both sets of field tests implied the copper filters suppressed nitrate measurements—most likely due to interference with the assay chemistry. All wet chemical nitrate sensors utilize the same chemical assay in which nitrate (NO_3_^–^) is first reduced to nitrite (NO_2_^–^), followed by the Griess assay, where the nitrite reacts with sulfanilamide to generate a positively charged diazonium intermediate which then couples with *N*-naphthyl-ethylenediamine (NEDD) to form a purple-colored product with absorbance maximum at 540 nm (Scheme 1).^26−28^ There are several reports of interference for measurements of soil extracts with high iron concentrations (10s to 100s of mg/L).^29−32^ This has been attributed to the reduction of the positively charged diazonium intermediate by Fe(II) ions.^29^ Given copper ions are of similar size and have similar divalent redox chemistry, it is therefore reasonable to expect a similar interference mechanism with copper cations.

**Figure 3:**
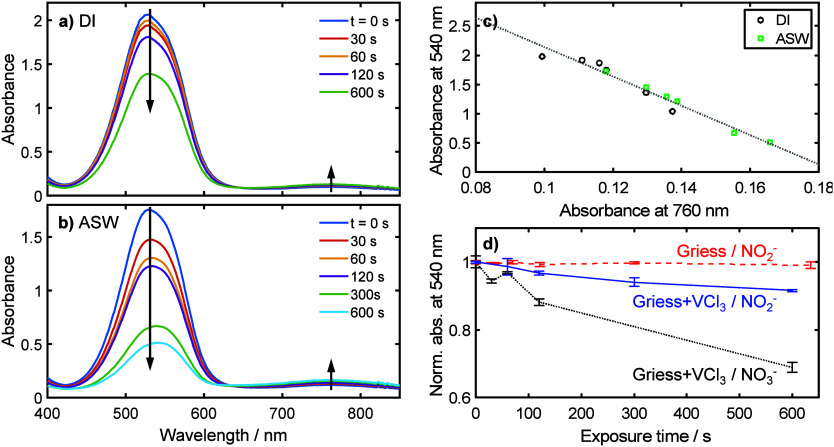
Replication of Cu interference under laboratory conditions. a) Absorbance spectra generated from water samples composed of nitrate in deionized (DI) water which had been exposed to Cu mesh for up to 10 min. Longer exposure times reduced the color generated by the Griess assay and gave slight increases at longer wavelengths. b) Absorbance spectra generated from water samples composed of nitrate in artificial seawater (ASW) which had been exposed to Cu mesh for up to 10 min. More pronounced trends are seen compared to DI water (a). c) Plot showing the negative correlation between absorbance at 540 nm (main Griess assay peak) and the absorbance at 760 nm in plots (a) and (b). d) Plot showing how the decrease in peak absorbance with increasing Cu exposure times was different for three different reagent/sample combinations: Griess without reductant and NO_2_^–^ (blue squares and dashed line), Griess with VCl_3_ reductant and NO_3_^–^ (orange circles and solid line), and Griess with VCl_3_ reductant and NO_3_^–^ (yellow triangles with dotted line). Error bars correspond to standard deviations from triplicate measurements.

To confirm this, we first looked to replicate the interference in a laboratory setting using the assay in isolation from a sensor. To do so we took samples of 300 μM NO_3_^–^ and placed them in contact with copper mesh for varying times (the same copper mesh used in the UoS filter) and subsequently analyzed them using the UoS version of the assay (which uses VCl_3_ as reductant) and a spectrophotometer (i.e., the same underlying colorimetric analysis method used in the sensor). Figure 3a shows the absorbance spectra obtained using nitrate solution made up in ultrapure deionized water (DI) that had been exposed to mesh for different times. Each sample exposed to the copper mesh exhibited a decrease in the main assay absorbance peak. With the exception of the 60 s measurement (which was slightly higher than the preceding 30 s measurement) longer exposure times generally led to a greater decrease in absorbance. When the experiment was repeated with artificial seawater (Figure 3b) the same trend was again observed but with more pronounced interference. Control experiments using other materials from the UoS filter displayed no such reduction in the Griess absorbance peak (data not shown), confirming the copper to be the source of the interference. ICP-OES elemental analysis of water samples exposed to the copper mesh confirmed the presence of copper ions (Supplementary Figure S1). Higher concentrations were found in artificial seawater compared to deionized water (11.4 μM vs 5.2 μM after 10 min exposure)—consistent with the higher electrolytic capacity and associated corrosion, and the increased interference in artificial seawater being due to increased dissolved copper concentrations.

Interestingly, the decrease in the main Griess peak was accompanied by the emergence of a peak centered at 760 nm (Figure 3a and 3b). The growth of this peak was strongly correlated with the reduction of the main absorbance at 540 nm, with a notably linear trend, as shown in Figure 3c. The position of the 760 nm absorbance corresponds to the absorbance peak for V(IV) aqua ions^33−35^ suggesting that the interference mechanism is not simply restricted to the diazonium intermediate but also impacts the initial nitrate to the nitrite reduction step by oxidizing the V(III) reductant. To further confirm this we repeated the test in DI water with two variations: first substituting nitrite for nitrate—meaning the VCl_3_ reductant is not part of the assay’s reaction pathway but is still present in the reaction solution—and second using nitrite instead of nitrate and removing the VCl_3_ from the reagent. Figure 3d shows the magnitude of the main peak for each case, normalized with respect to the absorbance obtained without copper mesh. Swapping nitrate for nitrite gave a dramatic reduction in interference, confirming that copper also interferes in the reduction step. That the interference was not removed completely, however, indicates that the copper also interferes with the subsequent Griess reaction as previously proposed. Removing the VCl_3_, however, completely removed the interference, indicating that the reductant was necessary for interference with the Griess reaction.

Literature reports of iron interference in soil nitrate analysis state that it is reduced iron, Fe(II), that is responsible for the interference.^29^ Given that the copper interference was observed in both the reduction step and the subsequent Griess reaction, these results imply that copper from the filters was initially in solution in its oxidized Cu(II) form (the predominant oxidation state of copper under aqueous conditions^36^) and then reduced by the vanadium reductant to yield Cu(I) which could subsequently interfere in the Griess reaction. The reduction of Cu(II) to Cu(I) will oxidize V(III) to V(IV), thus lowering the concentration of the reductant and reducing the nitrate reduction efficiency. Reduction of the copper by the vanadium is also consistent with the literature half-cell potentials: The V(IV)/V(III) reduction potential under acidic conditions, VO^2+^ + 2H^+^ + e^–^ → V^3+^, is +0.34 V;^33^ the standard Cu(II)/Cu(I) potential is +0.16 V,^37^ but under chloride rich conditions such as we have here (with both the vanadium salt and hydrochloric acid in the reagent contributing chloride), the Cu(II)/Cu(I) potential can be between 0.3 and 0.5 V higher^37,38^ reflecting the stabilizing effect of chloride on the Cu(I) ion;^36^ thus there is an overall favorable reaction potential between +0.12 and +0.32 V.

The implication of Cu(I) interference in the Griess reaction (after nitrate reduction) is also consistent with literature, with many reports of synthetic organic chemists having exploited the reaction of Cu(I) species with diazonium compounds as a strategy for generating functionalized aryl compounds. Referred to as the “Sandmeyer” reaction, the copper has been described as a catalyst that enables the replacement of the diazo group with a halide.^39−42^ As Cu(I) acts as a catalyst (and hence is not consumed during the reaction), then here a small amount of Cu(I) will have a disproportionate effect on the amount of product removed and hence nitrate measured.

**Figure 4:**
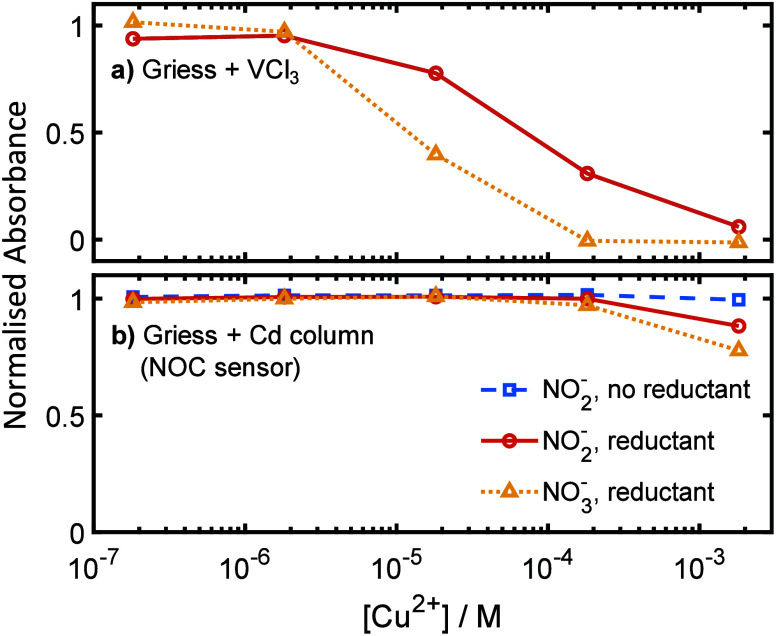
Effect of Cu(II) spiking. Normalized absorbance of the Griess assay for different concentrations of Cu(II) in the sample. a) Carried out using the UoS assay which uses VCl_3_ as reductant. b) Carried out using the NOC sensor, which includes a copperized Cd column as reductant. All absorbance values are normalized relative to the absorbance obtained with no copper present. Data points are mean values of triplicate measurements (standard deviation of measurements <2% in all cases).

If the VCl_3_ reductant was generating Cu(I), then we would expect similar results when the sample was spiked with a Cu(II) solution. To confirm this, and to also investigate the relation between interference and copper concentration, we measured the absorbance generated by a 300 μM sample of nitrite or nitrate spiked with different concentrations of copper(II) chloride. As shown in Figure 4a, the Cu(II) interfered in a similar way to the previous experiments using copper mesh, with interference greater when analyzing nitrate (yellow triangle markers) compared to nitrite (red circles). Increasing copper concentration increased interference for concentrations above a threshold of 2 μM, with the nitrate absorbance completely removed above 200 μM. The magnitude of the absorbance drop suggests that, in previous lab testing and UoS sensor field testing, the copper mesh generated Cu(II) concentrations in the order of 10 μM. It is interesting to note that this concentration is approximately 3 orders of magnitude lower than the concentration of the VCl_3_ reductant in the reagent (32 mM), which would initially seem to be inconsistent with the observed interference effect (how does a relatively small amount of copper have a disproportionate effect on the nitrate reduction step?), but it is worth noting that Cu(I) is typically unstable under aqueous conditions and is easily oxidized back to the more stable Cu(II).^36^ As such, Cu(I) will be short-lived and will be oxidized to Cu(II) where it can be reduced again.

At this point, lab testing had only used the homogeneous vanadium-based reduction used by the UoS sensor. The NOC sensor uses a very different reduction mechanism, however—heterogeneous reduction using a solid copperized cadmium catalyst. To ascertain whether the results from the vanadium assay were applicable to the different reduction method, the testing with samples spiked with Cu(II) (Figure 4a) was repeated using a NOC nitrate sensor (Figure 4b). As was the case for the vanadium-based assay, nitrate showed increased interference compared to nitrite, and no interference was seen in the absence of the reductant—confirming the same underlying interference mechanism. However, the sensitivity of the interference was notably different. Interference was only observed at mM concentrations, 2–3 orders of magnitude higher than the vanadium-based reduction method. The much lower sensitivity of the NOC sensor to the Cu(II) contrasts to the greater interference effect seen in the original field tests (Figure 2) and implies that the interference mechanism is different from the UoS—likely due to the different reductant (copperized cadmium column vs VCl_3_). Indeed, the reduction potential for copperized cadmium is +0.74 V,^22^ meaning we would not expect Cu(II) to be reduced by the cadmium column. Consequently if Cu(I) was not being generated *in situ* by the reductant, it must have entered the sensor in a stable form.

**Figure 5:**
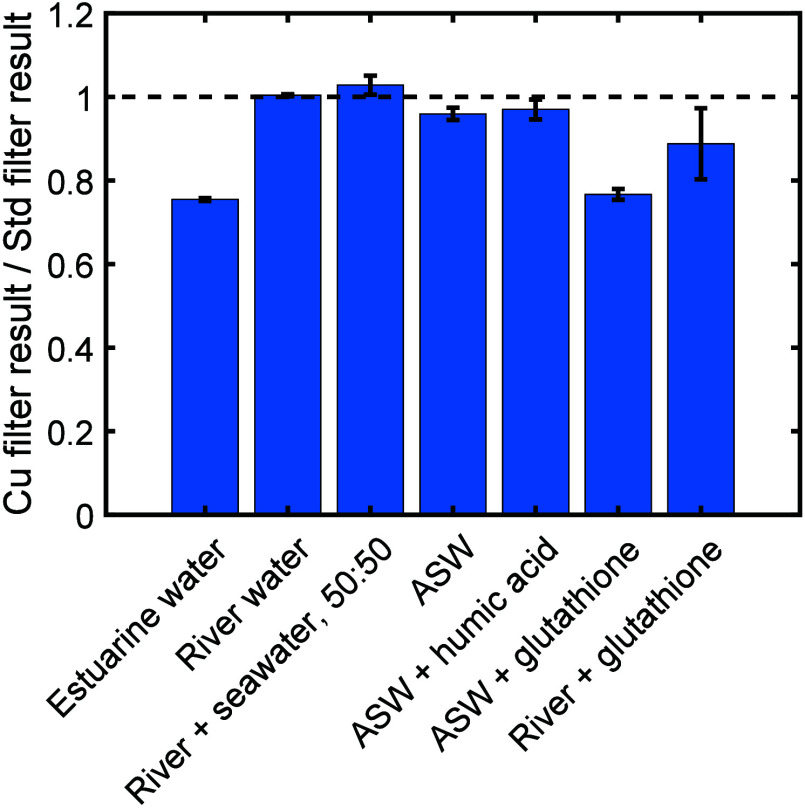
Interference observed when using estuarine water, river water, a 50:50 mix of river and seawater, artificial seawater (ASW), ASW with added humic acid, ASW with added glutathione, and river water with added glutathione.

A key clue as to how stable Cu(I) might be entering the sensor was found when trying to replicate the *in situ* copper filter interference using the NOC sensor in the laboratory. When measurements made using a copper filter were compared with those made with a standard filter (Figure 5), estuarine dock water taken from the same location as the initial field test gave interference similar to that originally seen *in situ*. Interference was not seen however when using river water (taken approximately 9 km upstream in the nontidal River Itchen), nor when it was mixed 50:50 with OSIL standard seawater. Interference was also not seen when using the standard seawater by itself (data not shown), or artificial seawater. That the estuarine water was the only sample to exhibit interference was initially surprising; however, this relates well to previous reports which describe how estuaries are notable environments for finding stabilized Cu(I)^43,44^ due to high concentrations of copper-chelating ligands, such as humic substances^45^ and thiols,^44,46^ combined with high salinity which further stabilizes Cu(I)^43^ due to the stability of Cu(I)-chloride complexes.^36,43^

To test whether ligands present in the estuarine water might be stabilizing the copper ions coming from the filter as Cu(I) we used a NOC sensor to measure an artificial seawater standard spiked with humic acid or glutathione (selected as a typical thiol found in estuarine water^46^). Humic acid can be found in estuaries at mg/L concentrations or higher,^47,48^ so humic acid was added at 8 mg/L and this concentration was also used for the glutathione for direct comparison. While the humic acid presented negligible interference, glutathione showed strong interference comparable to that shown by the estuarine water sample (Figure 5). Furthermore, when the same glutathione concentration was added to the river water sample, a notable interference was seen but at a reduced level, consistent with our previous observations that higher salinity increased interference (Figure 2) and previous observations that salinity also plays a key role in Cu(I) stabilization in estuarine environments.^43^

## Discussion

**Scheme 2:**
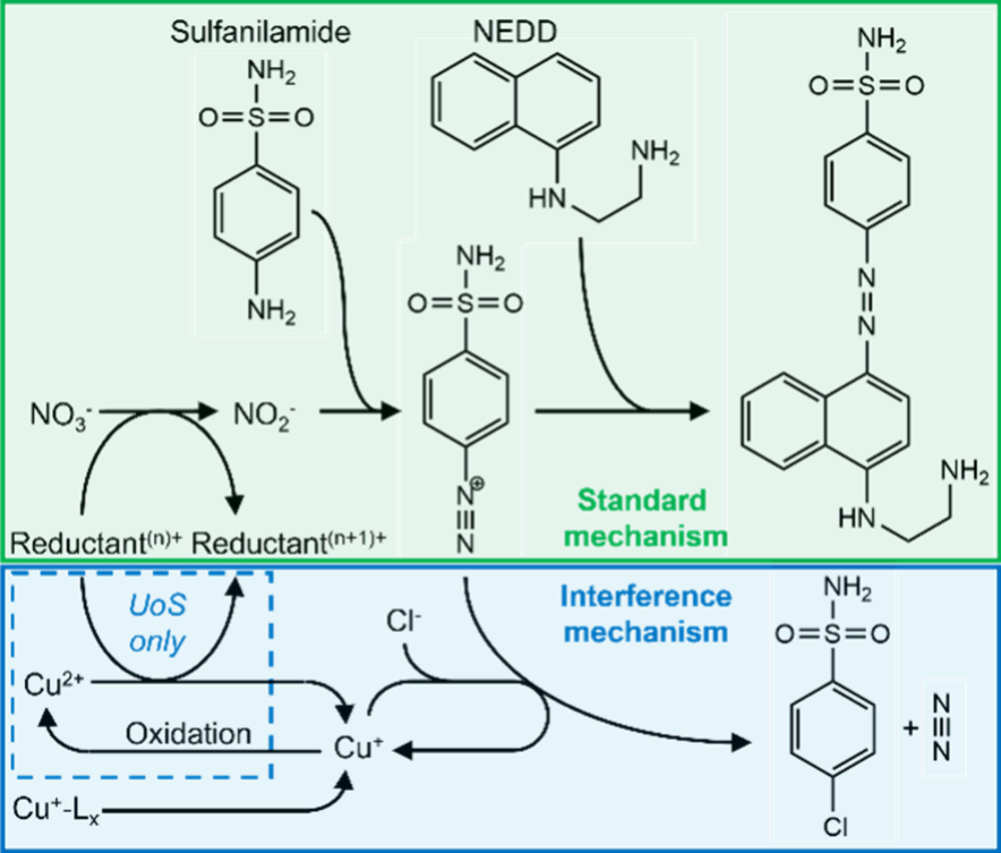
Standard Mechanism for the Nitrate Assay (Green Box, Top) Shown beside the Proposed Interference Mechanism The mechanism shown in the dashed box will only apply when using a reductant that can reduce Cu(II) to Cu(I) (such as the VCl_3_ used in the UoS sensor). Ligand-stabilized Cu(I) is shown as Cu^+^-L_*x*_.

The various laboratory tests point toward the interference mechanism proposed in Scheme 2. Copper, introduced into the sample by the filter, is initially present both in its more stable Cu(II) form and, depending on water composition, as Cu(I) stabilized by organic ligands. In the UoS sensor, the vanadium reductant can reduce Cu(II) to Cu(I). In doing so it is itself oxidized, thus reducing the amount of reductant and the reaction rate of the nitrate reduction. Examination of the half-cell potentials suggests that reduction of Cu(II) by VCl_3_ is only possible due to the chloride-rich reagent perturbing the standard Cu(II)/Cu(I) potential^37,38^ (overall reaction potential being an unfavorable −0.18 V when calculated using the standard potential but +0.12 V to +0.32 V allowing for chloride perturbation). The high concentrations of chloride in the reagent come from both hydrochloric acid (∼0.7 M) and vanadium chloride (0.1 M Cl^–^); hence substitution of these reagents for equivalents without halides (e.g., phosphoric instead of hydrochloric acid^49^) could be a viable route to reduce the interference. It should be noted that the droplet flow regime used in the UoS sensor requires homogeneous reagents; hence this would be a more feasible route compared to using the cadmium- or zinc-based heterogeneous reductants often used in the laboratory.^22,50^

As well as reduction from Cu(II), Cu(I) can also be naturally present if the incoming water contains suitable stabilizing ligands. We showed that the presence of glutathione (a typical thiol found in estuarine waters^46^) reproduced the interference experienced during *in situ* deployment; however, a range of different thiols and chelating species could play a similar role. Chemical additives that destabilize Cu(I) complexes and favor Cu(II) complexation could be used in the reagent as a potential strategy to tackle this interference source.

During the Griess reaction, Cu(I) is free to react with the diazonium intermediate in the Griess reaction to remove the diazo group and replace it with a substituent. While our testing does not tell us what that substituent is, as the reaction is commonly used in synthetic organic chemistry for addition of halides^51^ and as chloride ions are present in high concentration in the acidified Griess reagent, it is likely that chloride will be the substituent (see Scheme 2).

As already noted, the presence of chloride has an important role in the interference mechanism, and it also increases the amount of copper corroding off the solid copper filters (as confirmed by ICP results) while also stabilizing copper ions in the reduced Cu(I) form.^36,43^ These effects contribute to the increased salinity exacerbating the interference effect (see Figure 1a and Figure 2).

It is important to note that this copper interference results from the high concentrations of copper coming specifically from the filters. In the NOC sensor, it is unlikely to result from the solid copper on the surface of the copperized cadmium column. There the copper acts as a catalyst that accelerates electron transfer between the solid cadmium and molecules in solution.^22^ While the copper will accelerate a redox reaction (such as reduction of nitrate), it will not experience a net change in oxidation state; this instead happens to the cadmium which has the stronger reduction potential. As such, we would not expect redox reactions (e.g., corrosion) to perturb and remove copper, as any oxidation will preferentially occur on the cadmium, similar to the anticorrosion mechanism in galvanized steel.

Also, the high copper concentrations are not likely to ever result from ambient concentrations in natural waters. Testing showed interference occurred at Cu concentrations >1 μM (Figure 4)—much higher than reported natural concentrations where, for example, total Cu concentrations in European rivers are 15–140 nM^52,53^ and dissolved (<0.2 μm) Cu concentrations in north Pacific seawater have been reported at 1.0–3.5 nM.^54^ While suggestions have been made as to how this interference can be ameliorated, the most effective way to remove it would be to use other antifouling strategies such as regularly replacing filters, or using micropatterned surfaces.^55,56^ Nonetheless copper could still be a viable option for other wet chemical sensors that employ chemical assays insensitive to dissolved copper.

## Conclusions

Overall this work emphasizes how *chemical* antifouling strategies are part of the overall sensor measuring system, and therefore it is necessary to think holistically and consider how they will affect the overall operation of the sensor. More generally, this holistic approach should be applied when adapting any aspect of a sensor. Continuous data validation and establishment of best practices must be at the core of sensor use within the upcoming digital age of environmental science.

## Supporting Information Available

Experimental details describing the formulation of reagents and standards and ICP-OES experimental results (PDF)

## References

[ref1] Daniel A.; Laës-Huon A.; Barus C.; Beaton A. D.; Blandfort D.; Guigues N.; Knockaert M.; Munaron D.; Salter I.; Woodward E. M. S. Toward a Harmonization for Using in situ Nutrient Sensors in the Marine Environment. Frontiers in Marine Science 2020, 6, 773 10.3389/fmars.2019.00773.

[ref2] Martini M.; Butman B.; Mickelson M. J. Long-Term Performance of Aanderaa Optodes and Sea-Bird SBE-43 Dissolved-Oxygen Sensors Bottom Mounted at 32 m in Massachusetts Bay. Journal of Atmospheric and Oceanic Technology 2007, 24 (11), 1924–1935. 10.1175/JTECH2078.1.

[ref3] Venkatesan R.; Kadiyam J.; SenthilKumar P.; Lavanya R.; Vedaprakash L. Marine biofouling on moored buoys and sensors in the Northern Indian Ocean. Marine Technology Society Journal 2017, 51 (2), 22–30. 10.4031/MTSJ.51.2.11.

[ref4] Lehaitre M.; Delauney L.; Compère C. Biofouling and underwater measurements. In Real-time Coastal Observing Systems for Marine Ecosystem Dynamics and Harmful Algal Blooms: Theory, Instrumentation and Modelling; Babin M., Roesler C. S., Cullen J., Eds.; Oceanographic Methodology series; Unesco: 2008; pp 463–493.

[ref5] Yebra D. M.; Kiil S.; Dam-Johansen K. Antifouling technology—past, present and future steps towards efficient and environmentally friendly antifouling coatings. Prog. Org. Coat. 2004, 50 (2), 75–104. 10.1016/j.porgcoat.2003.06.001.

[ref6] MacIntyre G.; Plache B.; Lewis M.; Andrea J.; Feener S.; McLean S.; Johnson K.; Coletti L.; Jannasch H. ISUS/SUNA nitrate measurements in networked ocean observing systems. OCEANS 2009; IEEE: 2009; pp 1–7.

[ref7] Kerr A.; Smith M. J.; Cowling M. J.; Hodgkiess T. The biofouling resistant properties of six transparent polymers with and without pre-treatment by two antimicrobial solutions. Materials & Design 2001, 22 (5), 383–392. 10.1016/S0261-3069(00)00093-5.

[ref8] Chapman J.; Lawlor A.; Weir E.; Quilty B.; Regan F. Phthalate doped PVC membranes for the inhibition of fouling. J. Membr. Sci. 2010, 365 (1), 180–187. 10.1016/j.memsci.2010.09.003.

[ref9] Chavez F. P.; Wright D.; Herlien R.; Kelley M.; Shane F.; Strutton P. G. A device for protecting moored spectroradiometers from biofouling. Journal of Atmospheric and Oceanic Technology 2000, 17 (2), 215–219. 10.1175/1520-0426(2000)017<0215:ADFPMS>2.0.CO;2.

[ref10] Manov D. V.; Chang G. C.; Dickey T. D. Methods for reducing biofouling of moored optical sensors. Journal of Atmospheric and Oceanic Technology 2004, 21 (6), 958–968. 10.1175/1520-0426(2004)021<0958:MFRBOM>2.0.CO;2.

[ref11] Patil J. S.; Kimoto H.; Kimoto T.; Saino T. Ultraviolet radiation (UV-C): a potential tool for the control of biofouling on marine optical instruments. Biofouling 2007, 23 (4), 215–230. 10.1080/08927010701275598. 17653932

[ref12] McQuillan J. S.; Morris A. K.; Arundell M.; Pascal R.; Mowlem M. C. The anti-bacterial effect of an electrochemical anti-fouling method intended for the protection of miniaturised oceanographic sensors. J. Microbiol. Methods 2017, 141, 63–66. 10.1016/j.mimet.2017.08.006. 28803789

[ref13] Beaton A. D.; Cardwell C. L.; Thomas R. S.; Sieben V. J.; Legiret F.-E.; Waugh E. M.; Statham P. J.; Mowlem M. C.; Morgan H. Lab-on-chip measurement of nitrate and nitrite for in situ analysis of natural waters. Environ. Sci. Technol. 2012, 46 (17), 9548–9556. 10.1021/es300419u. 22835223

[ref14] Beaton A. D.; Sieben V. J.; Floquet C. F.; Waugh E. M.; Bey S. A. K.; Ogilvie I. R.; Mowlem M. C.; Morgan H. An automated microfluidic colourimetric sensor applied in situ to determine nitrite concentration. Sens. Actuators, B 2011, 156 (2), 1009–1014. 10.1016/j.snb.2011.02.042.

[ref15] Nightingale A. M.; Hassan S.-u.; Warren B. M.; Makris K.; Evans G. W.; Papadopoulou E.; Coleman S.; Niu X. A droplet microfluidic-based sensor for simultaneous in situ monitoring of nitrate and nitrite in natural waters. Environ. Sci. Technol. 2019, 53 (16), 9677–9685. 10.1021/acs.est.9b01032. 31352782

[ref16] Birchill A. J.; Clinton-Bailey G.; Hanz R.; Mawji E.; Cariou T.; White C.; Ussher S.; Worsfold P.; Achterberg E. P.; Mowlem M. Realistic measurement uncertainties for marine macronutrient measurements conducted using gas segmented flow and Lab-on-Chip techniques. Talanta 2019, 200, 228–235. 10.1016/j.talanta.2019.03.032. 31036178

[ref17] Vincent A. G.; Pascal R. W.; Beaton A. D.; Walk J.; Hopkins J. E.; Woodward E. M. S.; Mowlem M.; Lohan M. C. Nitrate drawdown during a shelf sea spring bloom revealed using a novel microfluidic in situ chemical sensor deployed within an autonomous underwater glider. Marine Chemistry 2018, 205, 29–36. 10.1016/j.marchem.2018.07.005.

[ref18] Yücel M.; Beaton A. D.; Dengler M.; Mowlem M. C.; Sohl F.; Sommer S. Nitrate and Nitrite Variability at the Seafloor of an Oxygen Minimum Zone Revealed by a Novel Microfluidic In-Situ Chemical Sensor. PLoS One 2015, 10 (7), e0132785 10.1371/journal.pone.0132785. 26161958 PMC4498834

[ref19] Beaton A. D.; Wadham J. L.; Hawkings J.; Bagshaw E. A.; Lamarche-Gagnon G.; Mowlem M. C.; Tranter M. High-resolution in situ measurement of nitrate in runoff from the Greenland ice sheet. Environ. Sci. Technol. 2017, 51 (21), 12518–12527. 10.1021/acs.est.7b03121. 28954516

[ref20] Walker D. Biofouling and its control for in situ lab-on-a-chip marine environmental sensors. University of Southampton, 2012.

[ref21] Evans G. W. H.; Nightingale A. M.; Hassan S.; Coleman S.; Niu X. A drop in the ocean: Monitoring of water chemistry using droplet microfluidics. Proceedings of MicroTAS 2017 2017.

[ref22] Zhang J.-Z.; Fischer C. J.; Ortner P. B. Comparison of Open Tubular Cadmium Reactor and Packed Cadmium Column in Automated Gas-Segmented Continuous Flow Nitrate Analysis. International Journal of Environmental Analytical Chemistry 2000, 76 (2), 99–113. 10.1080/03067310008034123.

[ref23] Nightingale A. M.; Evans G. W. H.; Xu P. X.; Kim B. J.; Sammer-ul H.; Niu X. Z. Phased peristaltic micropumping for continuous sampling and hardcoded droplet generation. Lab Chip 2017, 17 (6), 1149–1157. 10.1039/C6LC01479H. 28217768

[ref24] Nightingale A. M.; Hassan S.-u.; Evans G. W. H.; Coleman S. M.; Niu X. Nitrate measurement in droplet flow: gas-mediated crosstalk and correction. Lab Chip 2018, 18 (13), 1903–1913. 10.1039/C8LC00092A. 29877549

[ref25] Beaton A. D.; Schaap A. M.; Pascal R.; Hanz R.; Martincic U.; Cardwell C. L.; Morris A.; Clinton-Bailey G.; Saw K.; Hartman S. E.; Mowlem M. C. Lab-on-Chip for In Situ Analysis of Nutrients in the Deep Sea. ACS Sensors 2022, 7 (1), 89–98. 10.1021/acssensors.1c01685. 35020365

[ref26] Shinn M. B. Colorimetric Method for Determination of Nitrate. Industrial & Engineering Chemistry Analytical Edition 1941, 13 (1), 33–35. 10.1021/i560089a010.

[ref27] Patey M. D.; Rijkenberg M. J. A.; Statham P. J.; Stinchcombe M. C.; Achterberg E. P.; Mowlem M. Determination of nitrate and phosphate in seawater at nanomolar concentrations. TrAC Trends in Analytical Chemistry 2008, 27 (2), 169–182. 10.1016/j.trac.2007.12.006.

[ref28] Griess P.; Bemerkungen zu der Abhandlung der H. H. Weselsky und Benedikt “Ueber einige Azoverbindungen”. Berichte der deutschen chemischen Gesellschaft 1879, 12 (1), 426–428. 10.1002/cber.187901201117.

[ref29] Colman B. P. Understanding and eliminating iron interference in colorimetric nitrate and nitrite analysis. Environmental monitoring and assessment 2010, 165 (1–4), 633–641. 10.1007/s10661-009-0974-x. 19496004

[ref30] Colman B. P.; Fierer N.; Schimel J. P. Abiotic nitrate incorporation in soil: is it real?. Biogeochemistry 2007, 84 (2), 161–169. 10.1007/s10533-007-9111-5.

[ref31] Davidson E. A.; Dail D. B.; Chorover J. Iron interference in the quantification of nitrate in soil extracts and its effect on hypothesized abiotic immobilization of nitrate. Biogeochemistry 2008, 90 (1), 65–73. 10.1007/s10533-008-9231-6.

[ref32] Yang W. H.; Herman D.; Liptzin D.; Silver W. L. A new approach for removing iron interference from soil nitrate analysis. Soil Biology & Biochemistry 2012, 46, 123–128. 10.1016/j.soilbio.2011.12.003.

[ref33] Choi C.; Kim S.; Kim R.; Choi Y.; Kim S.; Jung H.-y.; Yang J. H.; Kim H.-T. A review of vanadium electrolytes for vanadium redox flow batteries. Renewable and Sustainable Energy Reviews 2017, 69, 263–274. 10.1016/j.rser.2016.11.188.

[ref34] Choi N. H.; Kwon S. K.; Kim H. Analysis of the Oxidation of the V(II) by Dissolved Oxygen Using UV-Visible Spectrophotometry in a Vanadium Redox Flow Battery. J. Electrochem. Soc. 2013, 160 (6), A973–A979. 10.1149/2.145306jes.

[ref35] Furman S. C.; Garner C. S. Absorption Spectra of Vanadium(III) and Vanadium(IV) Ions in Complexing and Non-complexing Media. J. Am. Chem. Soc. 1950, 72 (4), 1785–1789. 10.1021/ja01160a105.

[ref36] Cotton F. A.; Wilkinson G. Advanced Inorganic Chemistry; John Wiley & Sons Inc.: 1988.

[ref37] Lundström M.; Aromaa J.; Forsén O. Redox potential characteristics of cupric chloride solutions. Hydrometallurgy 2009, 95 (3), 285–289. 10.1016/j.hydromet.2008.06.009.

[ref38] Sanz L.; Palma J.; García-Quismondo E.; Anderson M. The effect of chloride ion complexation on reversibility and redox potential of the Cu(II)/Cu(I) couple for use in redox flow batteries. J. Power Sources 2013, 224, 278–284. 10.1016/j.jpowsour.2012.10.005.

[ref39] Galli C. Radical reactions of arenediazonium ions - an easy entry into the chemistry of the aryl radical. Chem. Rev. 1988, 88 (5), 765–792. 10.1021/cr00087a004.

[ref40] Sandmeyer T. Ueber die ersetzung der amidgruppe durch chlor in den aromatischen substanzen. Berichte der Deutschen chemischen Gesellschaft zu Berlin 1884, 17 (3), 1633–1635. 10.1002/cber.18840170219.

[ref41] Sandmeyer T. Ueber die ersetzung der amidgruppe durch chlor, brom und cyan in den aromatischen substanzen. Berichte der deutschen chemischen Gesellschaft 1884, 17 (4), 2650–2653. 10.1002/cber.188401702202.

[ref42] Kürti L.; Czakó B. Strategic applications of named reactions in organic synthesis: background and detailed mechanisms; Elsevier Academic Press: 2005.

[ref43] Buerge-Weirich D.; Sulzberger B. Formation of Cu(I) in Estuarine and Marine Waters: Application of a New Solid-Phase Extraction Method To Measure Cu(I). Environ. Sci. Technol. 2004, 38 (6), 1843–1848. 10.1021/es034845x. 15074698

[ref44] Whitby H.; Hollibaugh J. T.; van den Berg C. M. G. Chemical Speciation of Copper in a Salt Marsh Estuary and Bioavailability to Thaumarchaeota. Frontiers in Marine Science 2017, 4, 178 10.3389/fmars.2017.00178.

[ref45] Kogut M. B.; Voelker B. M. Strong Copper-Binding Behavior of Terrestrial Humic Substances in Seawater. Environ. Sci. Technol. 2001, 35 (6), 1149–1156. 10.1021/es0014584. 11347927

[ref46] Dryden C. L.; Gordon A. S.; Donat J. R. Seasonal survey of copper-complexing ligands and thiol compounds in a heavily utilized, urban estuary: Elizabeth River, Virginia. Marine Chemistry 2007, 103 (3), 276–288. 10.1016/j.marchem.2006.09.003.

[ref47] Boggs S. Jr; Livermore D.; Seitz M. G. Humic substances in natural waters and their complexation with trace metals and radionuclides: a review; 1985. 10.2172/5569909.

[ref48] Rodrigues A.; Brito A.; Janknecht P.; Proença M. F.; Nogueira R. Quantification of humic acids in surface water: effects of divalent cations, pH, and filtration. Journal of Environmental Monitoring 2009, 11 (2), 377–382. 10.1039/B811942B. 19212596

[ref49] Miranda K. M.; Espey M. G.; Wink D. A. A Rapid, Simple Spectrophotometric Method for Simultaneous Detection of Nitrate and Nitrite. Nitric Oxide 2001, 5 (1), 62–71. 10.1006/niox.2000.0319. 11178938

[ref50] Murray E.; Nesterenko E. P.; McCaul M.; Morrin A.; Diamond D.; Moore B. A colorimetric method for use within portable test kits for nitrate determination in various water matrices. Analytical Methods 2017, 9 (4), 680–687. 10.1039/C6AY03190K.

[ref51] Galli C. Substituent effects on the Sandmeyer reaction - quantitative evidence for rate-determining electron-transfer. Journal of the Chemical Society-Perkin Transactions 2 1984, (5), 897–902. 10.1039/p29840000897.

[ref52] Buykx S. E.; Cleven R. F.; Hoegee-Wehmann A. A.; van den Hoop M. A. Trace metal speciation in European river waters. Fresenius’ journal of analytical chemistry 1999, 363 (5–6), 599–602. 10.1007/s002160051257.

[ref53] Matthiessen P.; Reed J.; Johnson M. Sources and Potential Effects of Copper and Zinc Concentrations in the Estuarine Waters of Essex and Suffolk, United Kingdom. Mar. Pollut. Bull. 1999, 38 (10), 908–920. 10.1016/S0025-326X(99)00090-9.

[ref54] Whitby H.; Posacka A. M.; Maldonado M. T.; van den Berg C. M. G. Copper-binding ligands in the NE Pacific. Marine Chemistry 2018, 204, 36–48. 10.1016/j.marchem.2018.05.008.

[ref55] Cholkar A.; Chatterjee S.; Richards C.; McCarthy É.; Perumal G.; Regan F.; Kinahan D.; Brabazon D. Biofouling and Corrosion Protection of Aluminum Alloys Through Ultrafast Laser Surface Texturing for Marine Applications. Advanced Materials Interfaces 2024, 11 (6), 2300835 10.1002/admi.202300835.

[ref56] Richards C.; Ollero A. D.; Daly P.; Delauré Y.; Regan F. Disruption of diatom attachment on marine bioinspired antifouling materials based on Brill (Scophthalmus rhombus). Science of The Total Environment 2024, 912, 169348 10.1016/j.scitotenv.2023.169348. 38104837

