# Supplementary material for: Antifouling Copper Surfaces Interfere with Wet Chemical Nitrate Sensors: Characterization and Mechanistic Investigation
Source: ACS ES T Water. 2024 Dec 17;5(1):168–76. doi: 10.1021/acsestwater.4c00749 (PMC11731272; doi:10.1021/acsestwater.4c00749)
Supplement: Supplementary file 1 — ew4c00749_si_001.pdf [file ew4c00749_si_001.pdf]

Supplementary information for

Anti-fouling copper surfaces interfere with wet chemical nitrate sensors: characterisation and mechanistic investigation

Adrian M. Nightingale,[a]* Alexander D. Beaton,[b] Antony J. Birchill,[b] Sharon Coleman,[a] Gareth W. H. Evans,[a,c] Sammer-ul Hassan,[a,d] Matthew C. Mowlem,[b] & Xize Niu[a]

[a]Mechanical Engineering, Faculty of Engineering and Physical Sciences, University of Southampton, Southampton, SO17 1BJ, United Kingdom.

[b]Ocean Technology and Engineering Group, National Oceanography Centre, Southampton, SO14 3ZH, United Kingdom.

[c]Now at Lightcast Discovery Ltd, Broers Building, 21 JJ Thomson Ave, Cambridge CB3 0FA, United Kingdom.

[d]Now at Mechanical Engineering, Faculty of Engineering, The University of Hong Kong, China.

*a.nightingale@southampton.ac.uk

Supplementary Experimental Details

Chemicals

For the UoS assay, all chemicals were obtained at purity > 98% from Sigma Aldrich UK, except for vanadium(III) chloride (99.0 %) which was obtained from Alfa Aesar, UK and Fluorinert FC40 oil which was obtained from Acota Ltd, UK. Unless otherwise stated, water was ultra high purity (UHP) grade (18.2 MΩ.cm, Barnstead EASYpure RODI).

For all chemical work for the NOC sensor: Sodium chloride was obtained from VWR. Hydrogen carbonate, potassium nitrate, sulfanilamide, and imidazole were obtained from Fisher Scientific. Copper (II) sulfate, N-(1-Naphthyl)ethylenediamine dihydrochloride (NEDD), and chloroform were obtained from Sigma-Aldrich. Concentrated hydrochloric acid (37 %) was obtained from Fluka. All chemicals were obtained at purity > 98% and all reagent solutions and standards were made using ultra high purity (UHP) water (MilliQ >18.2 MΩ.cm, Millipore).

Griess reagents and standard solutions

UoS: The UoS's modified Griess reagent was formulated by first weighing out 1.25 g of vanadium(III) chloride and adding to a 250 ml volumetric flask along with 50 ml of ultrapure water to form a dark brown solution. 15 ml of concentrated (37 %) hydrochloric acid was added, causing the solution to turn a dark turquoise colour. 1.25 g of sulfanilamide and 0.125 g of NEDD was added, dissolved and the solution finally made up to the volumetric mark using ultrapure water.

A 100 mM stock solution of potassium nitrate was made by dissolving 2.528 g of potassium nitrate in ultrapure water in a 250 ml volumetric flask. A 100 mM stock solution of sodium nitrite was made by dissolving 1.725 g of sodium nitrite in ultrapure water in a 250 ml volumetric flask. The stock solutions were further diluted as required for each specific experiment.

A 10 mM stock solution of Cu(II) ions was formulated by adding 0.426 g of copper(II) chloride dihydrate to a 250 ml volumetric flask and making up with ultrapure water. This was then further diluted to make additional standard solutions with concentrations of 1000, 100, 10, and 1 µM.

NOC: For the NOC work Griess reagent (100 mL) was prepared by acidifying UHP water with hydrochloric acid (0.12 M), in which sulphanilamide (6 mM) and NEDD (0.54 mM) were dissolved. Imidazole buffer reagent (5000 mL) was prepared by dissolving imidazole (0.5 M), copper sulphate (10 μM) and hydrochloric acid (0.011 M) to achieve a pH of approximately 7.8.

Artificial seawater was made by dissolving sodium chloride (0.6 M) and hydrogen carbonate (8.2 mM) in UHP water.

A stock solution of copper(II) sulfate (10 mM) was made by dissolving salts in UHP water. For nitrate stock solutions potassium nitrate was dried in an oven at 105 °C for > 1 h and left to cool under vacuum in a desiccator. The dried salts were dissolved in UHP to give 5 mM solutions, which were sealed with parafilm to prevent evaporation. All stock solutions were stored in opaque HDPE bottles.

Working NO_3^- standards (100 mL) were prepared by diluting stocks in artificial seawater, preserved by the addition of chloroform (0.12 %). Blanks were prepared in the same manner but without the addition of NO_3^-.

Supplementary Figures

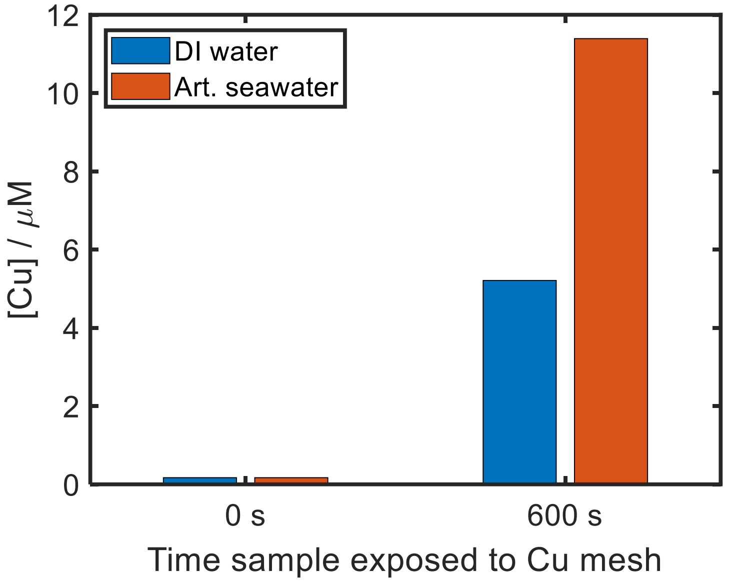

Figure S1: Copper concentrations within water samples exposed to copper mesh for different times. No copper is found in the samples before exposure, and after 600 s exposure copper is found in both samples, with twice as much in the artificial seawater compared to deionised (DI) water.
